# High Human Immunodeficiency Virus (HIV) Viral Load and Coinfection with Viral Hepatitis Are Associated with Liver Enzyme Abnormalities among HIV Seropositive Patients on Antiretroviral Therapy in the Lake Victoria Zone, Tanzania

**DOI:** 10.1155/2019/6375714

**Published:** 2019-06-02

**Authors:** Shabani Iddi, Caroline A. Minja, Vitus Silago, Asteria Benjamin, Jastine Mpesha, Shimba Henerico, Benson R. Kidenya, Stephen E. Mshana, Mariam M. Mirambo

**Affiliations:** ^1^Department of Physiology, Weill Bugando School of Medicine, Catholic University of Health and Allied Sciences, P.O. Box 1464, Mwanza, Tanzania; ^2^Department of Biochemistry and Molecular Biology, Weill Bugando School of Medicine, Catholic University of Health and Allied Sciences, P.O. Box 1464, Mwanza, Tanzania; ^3^Department of Microbiology and Immunology, Weill Bugando School of Medicine, Catholic University of Health and Allied Sciences, P.O. Box 1464, Mwanza, Tanzania; ^4^Bugando Medical Centre, P.O. Box 370, Mwanza, Tanzania

## Abstract

**Background:**

Liver enzymes abnormalities have been found to be common among patients on antiretroviral treatment (ART). Apart from the effects of ART on these changes, other factors that can potentially contribute to the abnormal levels of these enzymes have been found to vary in different geographical locations. This study investigated factors associated with liver enzymes abnormalities among human immunodeficiency virus (HIV) infected individuals on ART from the Lake Victoria zone, Tanzania.

**Methods:**

A cross-sectional study involving a total of 230 sera from HIV seropositive patients from different regions of the Lake Victoria zone was carried out in July 2017. All samples with required variables/parameters such as age, sex, ART regimen, and residence were serially included in the study. Hepatitis B virus (HBV) and Hepatitis C virus (HCV) detection and liver enzymes assays (alanine transaminase (ALAT) and aspartate transaminase (ASAT)) were assessed following the standard procedures. Data were analyzed by using STATA version 13.

**Results:**

The median age of the study participants was 38 (interquartile range [IQR]:30-48) years. The overall prevalence of abnormal liver enzymes was 43.04% (99/230, 95% CI: 36.6-49.3). A total of 26.09% (60/230) had elevated ASAT while 23.9% (55/230) patients had elevated ALAT levels. ASAT levels were significantly high among patients with high HIV viral load (P= 0.002) while ALAT levels were significantly high among those coinfected with hepatitis C virus (P=0.017) and hepatitis B virus (P<0.001).

**Conclusion:**

A significant proportion of HIV seropositive individuals on ART have abnormal levels of liver enzymes, which is significantly associated with high HIV viral load and viral hepatitis. This calls for the need to emphasize screening of viral hepatitis and provision of appropriate management among HIV seropositive individuals in this setting.

## 1. Background

In the era of highly active antiretroviral therapy (HAART), liver diseases have become one of the commonest nonacquired immune deficiency syndrome (AIDS) related causes of death. It accounts for 14–18% of mortalities among human immunodeficiency virus (HIV) infected patients [1, 2]. Among hospitalized HIV patients on HAART, almost half of deaths are attributed to liver diseases which range from asymptomatic or mild elevations of liver enzymes to cirrhosis, end stage liver disease, and other associated complications [3, 4]. Most of the studies have linked these abnormalities to hepatotoxicity caused by antiretroviral drugs without considering other factors such as HIV viral load and viral hepatitis among many others that can potentially cause abnormalities of the liver enzymes [5–7]. Several factors may be involved in these patients making it difficult to clearly establish the etiology. Concomitant infections with viral hepatitis such as hepatitis B (HBV) and hepatitis C (HCV), other opportunistic infections, alcohol abuse, AIDS related malignancies, and many other entities might be associated with abnormality of liver enzymes [8–10]. However, epidemiology and distribution of these factors might differ from different geographical locations with limited reports from low- and middle-income countries. This study aimed at determining factors associated with liver enzyme abnormalities among HIV population attending different centers in the Lake Victoria zone, Tanzania. The findings from this study might help the health care providers to establish proper monitoring and management of HIV infected individuals by increasing awareness about other factors that can contribute to the abnormalities of liver enzymes in this population.

## 2. Methods

### 2.1. Study Design, Area, and Period

This was a cross-sectional hospital based study which was conducted in July 2017 at the Bugando Medical Centre (BMC). BMC is a consultant and teaching hospital with about 900 bed capacity located in the Northwestern of Tanzania serving the lake zone regions, namely, Mwanza, Mara, Kagera, Shinyanga, Simiyu, and Kigoma with estimated population size of thirteen million people. BMC process samples for viral load for all CTCs in the lake zone and in each day samples from all centers are received.

### 2.2. Sample Size Estimation, Study Population, Sampling, and Selection Criteria

Sample size was estimated by using Kish Leslie formula using the prevalence of 16.4% [11]; the minimum sample size was 211 sera. However, a total of 230 samples were collected. All blood samples collected from different care and treatment centers (CTCs) in different regions for viral load testing at BMC were eligible to be included in the study. Samples with required variables/parameters (age, sex, residence, ART regimen (first, second, or third line), reason for HIV viral load testing (first test, repeating test, or suspected treatment failure), and drug adherence) were serially included until the required sample size was reached.

### 2.3. Data Collection Procedure

Sociodemographic and other relevant information such as age, sex, residence, ART regimen (first, second, or third line), reason for HIV viral load testing (first test, repeating test, or suspected treatment failure), and drug adherence (good, fair, or poor) were extracted from laboratory request forms accompanying samples for viral load testing using a checklist. Blood specimens in vacutainer EDTA tubes (BD, Franklin Lakes, New Jersey, USA) collected from different CTCs in different regions for viral load testing at BMC were used in this study.

### 2.4. Laboratory Procedures

EDTA blood samples were centrifuged at 4000 revolutions per minute for 20 minutes to obtain plasma which was then used for viral load testing. Viral load testing was done using a COBAS TaqMan analyzer (Roche diagnostics, Germany) following the manufacturer's instructions. Viral suppression was defined as viral load < 1000 copies/mL [12].

The liver enzymes (ASAT and ALAT) were analyzed using the CIBA CORNING 252 calorimeter (Ciba Corning Analytical Halstead, England). Liver enzymes were considered normal when values range from 2-40 IU/mL for ASAT and 2-41IU/mL for ALAT. In addition, all samples were analyzed for the presence of HBV antigens and HCV antibodies using immunochromatographic tests (Wondfo HBsAg and HCV antibodies, Guangzhou, China) following the manufacturer's instructions.

### 2.5. Quality Control

The standard operating procedures were strictly followed for the quality assurance. Control materials with known ALAT and ASAT results were used to calibrate the calorimeter as per assay kit instructions before processing the samples for ASAT and ALAT. HBV and HCV kits were quality checked using known HBsAg and anti-HCV antibody positive and negative sera controls.

### 2.6. Data Management and Analysis

Every sample was given a unique identification number. All data were recorded in the log book, transferred to excel then transferred to STATA version 13 (San Antonio, Texas) for cleaning and analysis. Results were presented into proportions for categorical variables and median (IQR) for continuous variables. Chi square test was used to test the association between liver enzymes abnormalities and other factors such as viral hepatitis and HIV viral load followed by multivariate logistic regression analysis to establish independent predictors. Wilcoxon Rank Sum Mann–Whitney test was used to compare median for various groups. A P value of < 0.05 at 95% confidence interval was considered statistically significant.

### 2.7. Ethical Considerations

Ethical clearance for using patient's samples was sought from the joint CUHAS/BMC research ethics and review committee with ethical clearance number CREC/381/2017. Permission to conduct the study was requested from hospital laboratory administration. All patient-related information was stored carefully and anonymously using codes.

## 3. Results

### 3.1. Sociodemographic and Clinical Data of the Participants

A total of 230 HIV seropositive individuals on ART with the median age of 33 (IQR: 30-48) years participated in this study. The slight majority of the participants 152 (66.1%) were females. A significant proportion of them were from Shinyanga (30.43%), Bunda (26.5%), and Butiama (21.3%). Other characteristics are shown in Table 1. The median age of participants with high ASAT levels was 24(IQR: 15-41) years while that of those with high ALAT levels was 33(IQR: 22-41) years. On Wilcoxon Rank Sum Mann–Whitney test, there was no significant difference on age among participants with high ASAT levels compared to their counterparts [38(IQR: 29.5-47) vs. 38(IQR: 30-48) years, P=0.577] while the median age of participants with high ALAT levels was significantly low compared to their counterparts [33(IQR: 27-43) vs. 39(IQR: 30-49), P=0.036].

### 3.2. Prevalence of Liver Enzyme Abnormalities and Associated Factors among HIV Infected Patients

Overall, the prevalence of liver enzyme abnormalities was 43.04% (99/230, 95% CI: 36.6-49.3). A total of 60 patients (26.09%) had elevated ASAT level while 55 patients (23.9%) had elevated ALAT levels (Table 1). Regarding ASAT levels, on univariate analysis high HIV viral load (P=0.001), coinfection with HBV (P<0.001), and HCV (P=0.017) were significantly associated with elevated ASAT levels. Only high HIV viral load (OR: 2.86, 95% CI:1.45-5.65, P=0.002) was independently found to predict high levels of ASAT (Table 2). On the other side coinfection with HCV was significantly associated with elevated ALAT levels (P=0.017) on univariate analysis while none of the factors was found to predict ALAT levels on multivariate logistic regression analysis (Table 3).

## 4. Discussion

Liver enzyme abnormalities are common among human immunodeficiency virus (HIV) infected individuals and have been associated with multiple factors. However, the risk factors associated with these abnormalities tend to differ in different geographical locations. To the best of our knowledge, this is the first study to investigate factors associated with liver enzyme abnormalities in the Lake Victoria zone, Tanzania. The most salient finding in this study is high overall prevalence of liver enzyme abnormalities which was observed to be 43.09%. This was significantly high in comparison to previous studies in Cameroon and South Africa which observed the prevalence of (22%) and (23%), respectively [13, 14]. Furthermore, in comparison with a previous study in Rwanda [11] in East Africa, the prevalence reported in this study is indeed significantly high. These variations could be explained by genetic variations in metabolizing antiretroviral drugs which may affect the drug toxicity. In addition, elevated liver enzymes in HIV infected patients might be due to direct inflammation of hepatocytes by HIV virus through apoptosis, mitochondrial dysfunction, and permeability alteration in mitochondrial membrane that stimulates an inflammatory response [15]. This has been further confirmed in this study whereby liver enzyme abnormalities were significantly associated with high HIV viral load.

In this study, liver enzyme abnormalities were found to be significantly associated with viral hepatitis (HBV and HCV); this observation was similar to the previous studies [13, 16–19]. In addition, Cicconi et al. observed high levels of liver enzyme abnormalities among HIV-viral hepatitis coinfected patients than HIV monoinfected patients [20]. The possible explanation could be viral cytopathic effects [21]. Further studies to investigate the HBV and HCV viral load in relation to liver enzymes abnormalities in HIV infected patients are of paramount importance.

Another observation in this study was significant association between high ALAT levels with young age. This could be explained by the fact that young aged individuals can mount strong immunity than old aged individuals; therefore, the elevated ALAT could be associated with immunopathology following viral infections [22].

## 5. Limitation of the Study

One of the major limitations of this study could be the sensitivity of the assays which might affect the results. In addition, other factors that can cause liver damage such as alcohol abuse and opportunistic infections were not investigated.

## 6. Conclusion

This study observed high prevalence of liver enzyme abnormalities which was significantly associated with viral hepatitis and high HIV viral load among HIV infected patients in the Lake Victoria zone, Tanzania. Therefore, monitoring and management of liver enzymes abnormalities in HIV infected patients are essential in this setting.

## Figures and Tables

**Table 1:** Demographic and clinical characteristics of HIV positive individuals in the lake zone (N=230).

Variables	Categories	Frequency (n)	Percent
*Age∗*		37.35(1-75)	

*Sex*	Female	152	66.09%
	Male	78	33.91%

*ART Regimen*	1g-A(TDF+3TC+EFV)	111	48.26
	1b-A(AZT+3TC+NVP)	40	17.39
	2H-A(TDF+FTC+ATV/R)	5	2.17
	2s-A (AZT+3TC+ATV/R)	1	0.43
	Ic-P(AZT+3TC+EFV)	4	1.74

*ASAT*	Normal	170	73.91
	High	60	26.09

*ALAT*	Normal	175	76.09
	High	55	23.91

Age*∗*	High ASAT	24(15-41)	
	High ALAT	33(22-41)	

**∗**: age (in years) in median (IQR).

*AZT* = Zidovudine, *EFV* = Efavirenz, *R* = Ritonavir, *NVP* = Nevirapine, *TDF*= Tenofovir, *3TC* = Lamivudine, *ATV* = Atazanavir, *FTC* = Emtricitabine.

**Table 2:** Factors associated with high ASAT levels among HIV positive individual in the lake zone (N=230).

		Univariate analysis			Multivariate analysis	
Variable	Categories	Normal ASAT (2 -40 IU/L)	High ASAT (>40 IU/L)	P-value	OR[95%CI]	P-value
		n(% )	n(%)			

Age*∗*			70(56-96)		0.99[0.96-1.01]	0.514

Sex	Female	115(75.66)	37(24.34)			
	Male	55(70.51)	23(29.49)	0.400		

IgA	No	84(70.59)	35(29.41)	0.234		
	Yes	86(77.48)	25(23.52)			

IbA	No	145(76.32)	45(26.68)			
	Yes	25(62.50)	15(37.50)	0.071	1.4(0.6-3.5)	0.367

IcA	No	138(73.80)	49(26.20)	0.933		
	Yes	32(74.42)	11(25.58)			

IeA	No	144(73.10)	53(23.90)	0.491		
	Yes	26(78.79)	7(21.21)			

Adherence	Good	87(79.09)	23(20.01)			
	Poor	83(69.87)	37(30.83)	0.087	1.39[0.68-2.80]	0.357

Viral load	Suppression	126(80.25)	31(19.75)			
	No suppression	44(60.27)	29(39.73)	*0.001∗∗*	2.86[1.45-5.65]	*0.002∗∗*

HBV	Negative	170(77.98)	48(22.02)			
	Positive	0(0.00)	12(100)	*<0.001∗∗*		

HCV	Negative	170(74.56)	58(25.44)			
	Positive	0(0.00)	2(100)	*0.017∗∗*		

*∗*: age (in years) in Median (IQR).

*∗∗*: significant association.

**Table 3:** Factors associated with high ALAT levels among HIV positive individual in the lake zone (N=230).

		Univariate regression			Multivariate regression	
Variable	Categories	Normal ALAT (2-41IU/L)	High ALAT (>41IU/L)	P-value	OR[95%CI]	P-value
		*n(%)*	*n(%)*			

Age*∗*			78(67-98)		0.98[0.96-1.00]	0.171

Sex	*Female*	116(76.32)	36(26.68)	0.910		
	*Male*	59(75.64)	19(25.36)			

IgA	No	91(76.47)	28(23.53)			
	Yes	84(75.68)	27(25.32)	0.888		

IbA	No	144(75.79)	46(24.21)	0.818		
	Yes	31(77.50)	9(22.50)			

IcA	No	142(75.94)	45(24.06)	0.911		
	Yes	33(76.74)	10(23.26)			

IeA	No	151(76.65)	46(23.35)			
	Yes	24(72.73)	9(27.27)	0.625		

Adherence	Good	89(80.91)	21(19.09)			

	Poor	86(71.67)	34(28.33)	0.101	1.61[0.81-3.18]	0.170

Viral	Suppression (<1,000 copies/mL)	125(79.62)	32(20.68)			
	No suppression (≥ 1,000 copies/mL)	50(68.49)	23(31.51)	0.066	1.75[0.88-3.49]	0.107

HBV	Negative	171(78.44)	47(21.56)			
	Positive	4(33.33)	8(66.67)			

HCV	Negative	170(74.56)	58(25.44)			
	Positive	0(0.00)	2(100)	*0.017∗∗*		

*∗*: age (in years) in Median (IQR).

*∗∗*: significant association.

## Data Availability

The data used to support the findings of this study are included in the article.
